# α7‐Nicotinic Acetylcholine Receptor and Mutated α‐Synuclein Interact in Motor Behaviour and Nigrostriatal Dopamine—Findings With Potential Relevance for a Protective Effect of Cigarette Smoking and Parkinson's Disease

**DOI:** 10.1111/ejn.70063

**Published:** 2025-03-24

**Authors:** Christian Pifl, Alexandra Wolf, Mark Elevado, Petra Scholze

**Affiliations:** ^1^ Department of Molecular Neurosciences, Center for Brain Research Medical University of Vienna Vienna Austria; ^2^ Department of Pathobiology of the Nervous System, Center for Brain Research Medical University of Vienna Vienna Austria

**Keywords:** alpha‐synuclein, nicotine, nicotinic acetylcholine receptor, Parkinson's disease

## Abstract

Parkinson's disease (PD) occurs less frequently in cigarette smokers than in nonsmokers. Assuming that nicotinic acetylcholine receptors are periodically active by activation through endogenous acetylcholine, we tested whether they act against the effect of α‐synuclein, a protein relevant in PD. Transgenic mice with a human α‐synuclein containing two mutations that cause familial PD were crossed with mice lacking the nicotinic α7‐acetylcholine receptor. Vertical movements determined at 7 and 16 months and nonambulatory movements at 16 months of age were significantly lower in mice with α7‐acetylcholine receptor knockout if they express the mutated α‐synuclein but not in mice with α‐synuclein wild type. Striatal noradrenaline, serotonin and dopamine levels did not differ between the four groups of mice at 21 months; however, striatal dopamine turnover was significantly higher in mice without than with α7‐acetylcholine receptor. Stereological counts of nigral cells positive for tyrosine hydroxylase in the left and right hemisphere at 21 months revealed that asymmetry was also significantly higher in mice without than with α7‐acetylcholine receptor. In conclusion, up to the age of 16 months, there was no obvious PD behaviour; however, absence of the α7‐acetylcholine receptor generally reduced several features of motor behaviour and showed a statistically significant interaction between α7‐acetylcholine receptor and mutated α‐synuclein. The asymmetry of nigral cell counts and the increased striatal dopamine turnover suggest a stressed nigrostriatal system in mice without α7‐acetylcholine receptor and that the neuroprotective effect of smoking might at least partly be mediated by the nicotine in the cigarettes acting via α7‐acetylcholine receptors.

## Graphical abstract

Mice with a mutated human α‐synuclein were crossed with mice lacking the nicotinic α7‐acetylcholine receptor; offsprings were analysed for signs of Parkinson's disease. Mice with mutated α‐synuclein without α7‐acetylcholine receptor had an asymmetry of nigral cell counts and an increased striatal dopamine turnover apart from some motor impairments. This suggests a stressed nigrostriatal system, and the neuroprotective effect of smoking might at least partly be mediated via α7‐receptors.

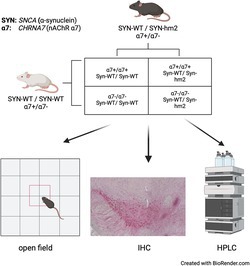

**Abbreviations**
5‐HIAA: 5‐hydroxyindoleacetic acid
5‐HT: serotonin
α7 nAChR: (pentameric) α7 nicotinic acetylcholine receptor
α7‐KO: mice lacking the *CHRNA7* gene, which codes for the α7 nAChR
DA: dopamine
DOPAC: 3,4‐dihydroxyphenylacetic acid
HPLC/ED: high‐performance liquid chromatography system with electrochemical detection
HV: homovanillic acid
MPTP: 1‐methyl‐4‐phenyl‐1,2,3,6‐tetrahydropyridin
NA: noradrenaline
PD: Parkinson's disease
Syn‐hm2: Transgenic mice heterozygous for a doubly mutated form of human α‐synuclein (A53T and A30P)

## 1 Introduction

Idiopathic Parkinson's disease is still of unknown aetiology. There is a range of environmental and genetic risk factors for the development of Parkinson's disease (Noyce et al. 2012; Nalls et al. 2019; Cherian et al. 2023), and according to numerous epidemiological studies, PD occurs less frequently in cigarette smokers than in nonsmokers (Hernan et al. 2002; Li et al. 2015). The higher the number of cigarettes smoked per day and the higher the number of years of smoking, the lower the odds to develop PD (Chen et al. 2010). Even for passive smokers, a reduced risk of Parkinson's disease has been reported (Searles Nielsen et al. 2012; Gallo et al. 2019). Although the negative association of Parkinson's disease with smoking was also explained by confounding factors such as an increased ability of patients with Parkinson's disease to quit smoking as compared to controls (Ritz et al. 2014), studies propose an association not explainable by reverse causation and a causally protective effect of current smoking (Domenighetti et al. 2022; Mappin‐Kasirer et al. 2020). A protective effect of cigarette smoke was related to its monoamine oxidase inhibiting effect, because 1‐methyl‐4‐phenyl‐1,2,3,6‐tetrahydropyridin (MPTP) depends on monoamine oxidase to a form an active agent that induces a parkinsonism nearly undistinguishable from idiopathic Parkinson's disease in human and subhuman primates (Castagnoli and Murugesan 2004), and a related, still undiscovered mechanism might be relevant in Parkinson's disease. Furthermore, the antiparkinsonian medication rasagiline with monoamine oxidase inhibiting properties provides not only symptomatic relief but might have neuroprotective properties as suggested by delayed need for antiparkinsonian drugs (Rascol et al. 2011). Mutation in RIC3, a chaperone of α7‐acetylcholine receptors, provides additional evidence for the role of cholinergic systems in Parkinson pathology (Sudhaman et al. 2016). Although cigarette smoke is a complex mixture of constituents, nicotine is the pharmacologically most prominent ingredient and the eponymous agonist of the nicotinic acetylcholine receptors. Already decades ago, a neuroprotective role of nicotinic acetylcholine receptors was demonstrated against the excitatory neurotoxicity of glutamate, one of the risk factors for neurodegenerative diseases (Akaike et al. 1994). Neuronal nicotinic acetylcholine receptors are pentameric ligand‐gated ion channels. Most nicotinic acetylcholine receptors are heteromeric assemblies of five partially distinct subunits; the α7 and the α9 subunits are the only mammalian subunits, which form homomeric, rather than heteromeric receptors (Zoli et al. 2018). Selective α7‐receptor agonists protected against β‐amyloid‐ and glutamate‐induced neuronal death in cultured rat cortical neurons (Kihara et al. 1997), and α7‐neuronal receptor antagonists blocked the protection by nicotine against glutamate‐induced cytotoxicity in rat cortical neurons (Kaneko et al. 1997) and rotenone‐induced Parkinson's disease models (Takeuchi et al. 2009). α7 nicotinic acetylcholine receptor stimulation protected against the dopaminergic neurotoxin 6‐hydroxydopamine in rats (Suzuki et al. 2013; Jiang et al. 2020). Randomized clinical trials with nicotine in patients with Parkinson's have not been very encouraging so far (Oertel et al. 2023; Vieregge et al. 2001; Villafane et al. 2018). On one hand, clinical trials, even if they start at an early disease‐stage, might still come too late, because a large number of dopamine‐containing cells have already been lost (Kalia and Lang 2015). In addition, the continuous nicotine administration via patch in these trials differs from pulsatile nicotine obtained by smoking in the dynamic of nicotine acetylcholine receptor stimulation. This is especially relevant, as nicotinic acetylcholine receptors get easily desensitized, in particular by the agonist nicotine (Pidoplichko et al. 1997; Albuquerque et al. 2009). The issue of nicotine administration and the contribution of nonreceptor‐mediated mechanisms of nicotine (Castagnoli and Murugesan 2004; Cormier et al. 2003; Xie et al. 2005) prompted us to investigate the protective effect of nicotine receptor stimulation using a completely different approach. We hypothesized that nicotinic α7‐acetylcholine receptors are neuroprotective even in the absence of nicotine exposure by being periodically active through stimulation by the neurotransmitter acetylcholine, and the absence of these α7‐receptors might reveal neurodegeneration by a genetic risk factor. As a genetic risk factor, we chose a double‐mutated form of human α‐synuclein reported to produce a locomotor phenotype of PD with changes in dopamine levels and turnover in mice overexpressing it under the tyrosine hydroxylase promotor (Richfield et al. 2002). We investigated the effect of the α7‐receptor subtype out of the numerous nicotinic receptor subtypes, because (1) it is one of the most abundantly expressed and widely distributed subtype in the brain (Gotti and Clementi 2004; Sargent 1993); (2) it is not only expressed in neurons but also in astrocytes and microglia, which might be relevant for Parkinson's disease (Liu et al. 2015; Suzuki et al. 2006; Soares et al. 2024); (3) there are reports on neuroprotective effects of the α7‐receptor subtype in Parkinson's disease relevant models (Takeuchi et al. 2009; Suzuki et al. 2013; Jiang et al. 2020); and (4) knockdown of the α7‐receptor subtype is a more straight‐forward change due to its predominance in homomeric pentamers than knockdown of subunits with occurrence in various forms of heteromeric nicotinic acetylcholine receptors.

## 2 Experimental Procedures

### 2.1 Breeding and Housing of Mice

Transgenic mice heterozygous for a doubly mutated form of human α‐synuclein (A53T and A30P, Syn‐hm2/Syn‐WT) under control of the 9‐kb rat tyrosine hydroxylase promotor (Richfield et al. 2002) were crossed with mice without nicotinic α7‐acetylcholine receptors (α7−/α7−) (Orr‐Urtreger et al. 1997) (Jackson Laboratory), which resulted in Syn‐hm2/Syn‐WT/α7+/α7− and Syn‐WT/Syn‐WT/ α7+/α7− mice. Crossing of these mice with each other resulted in the first experimental group Syn‐hm2/Syn‐WT/α7+/α7+ (=Syn‐hm2/α7‐WT) and Syn‐hm2/Syn‐WT/α7+/α7− mice. Crossing of Syn‐hm2/Syn‐WT/α7+/α7− with Syn‐WT/Syn‐WT/α7+/α7− mice resulted in Syn‐WT/Syn‐WT/α7+/α7− mice and the three other experimental groups of Syn‐hm2/Syn‐WT/α7−/α7− (=Syn‐hm2/ α7‐KO), Syn‐WT/Syn‐WT/α7+/α7+ (=Syn‐WT/ α7‐WT) and Syn‐WT/Syn‐WT/α7−/α7− (=Syn‐WT/α7‐KO) (the breeding scheme is also depicted in Table S1).

Animals were kept in thermo‐stable rooms (21°C) on a light/dark schedule of 10/14 h in group cages with food and water freely accessible. All animal experiments were in accordance with EU (Directive 210/63/EU) and Austrian federal law (Tierversuchsgesetz 2012) after obtaining approval from the animal subjects review board of the Medical University of Vienna and the Austrian Federal Ministry of Education, Science and Research (GZ: 2022‐0.638.952).

Genotyped male littermates of these four types of mice, Syn‐hm2/α7‐WT (*n* = 12), Syn‐hm2/α7‐KO (*n* = 14), Syn‐WT/α7‐WT (*n* = 19) and Syn‐WT/α7‐KO (*n* = 15) were housed in cages of four to six animals and tested for the motor features of ambulatory, nonambulatory and vertical movements at 7 and 16 months of age. At 21 months of age, the mice were decapitated; the brain was rapidly removed from the skull and placed in a mixture of 0.9% NaCl‐solution and ice of 0.9% NaCl and split into a rostral part to be frozen on dry ice and a brain stem part, which was postfixed in 4% paraformaldehyde in phosphate buffered saline to be finally embedded into paraffin.

### 2.2 Genotyping

Animals were genotyped by analysing ear punches using the KAPA HotStart Mouse Genotyping Kit (Merck). The presence of the doubly mutated form of human α‐synuclein was verified using the Primers CAG GTA CCG ACA GTT GTG GTG TAA AGG AAT and GAT AGC TAT AAG GCT TCA GGT TCG TAG TCT, leading to a 470 bp PCR product in transgenic mice, which is absent in nontransgenic animals (Richfield et al. 2002). α7 animals were analysed using a mix of three primers (CCT GGT CCT GCT GTG TTA AAC TGC TTC, CTG CTG GGA AAT CCT AGG CAC ACT TGA G, GAC AAG ACC GGC TTC CAT CC), resulting in a single PCR product of 440 bp in α7+/α7+, a single product of 750 bp in α7−/α7− and both products in α7+/α7− heterozygous animals (Orr‐Urtreger et al. 1997).

### 2.3 Measurement of Motor Behaviour

Motor behaviour was recorded in single mice by an activity metre (Optovarimex, Columbus Instruments, OH, United States) as described previously (Hinzen et al. 1986; Pifl and Hornykiewicz 1988). A grid of infrared photocells placed around an arena of 30 × 30 cm allowed differentiation of ambulatory movements, where the movement consists in a displacement, locomotor activity in the narrower sense, from nonambulatory movements without place change, a kind of stereotypic behaviour as found strongly increased by high doses of dopaminergic drug, such as compulsive head bobbing and grooming behaviour, calculated by a built‐in microprocessor from beam interruptions without body displacement. An additional array of photocells allowed quantification of vertical movements a kind of rearing anywhere in the open field. In order to minimize stress effects by taking out and bringing back mice into the home cage, the effort was made to test only one mouse from each cage per day. Movements accumulated in 5‐min intervals were shown in the figures over a period of 45 min after the mouse was placed in the activity cage.

### 2.4 Measurement of Monoamine Neurotransmitter and Dopamine Metabolites

Striatum was dissected on a −10°C cold plate from coronal slices of the frozen rostral part of the brain, ultrasonicated with an ultrasonic probe sonicator in 50 volumes of ice‐cold perchloric acid, sodium bisulphite and 3,4‐dihydroxybenzylamine as internal standard (final concentration 0.1 M, 0.4 mM and 50 μg/L, respectively). For determination of noradrenaline, homogenates were centrifuged at 16,100×*g* for 10 min at 4°C, and 167 μL of supernatant was extracted with alumina oxide (10 g/L) in 1‐M Tris–HCl, pH 8.6, and after washing with H_2_O and desorption with 0.2 mL 0.1 M perchloric acid containing 0.4 mM sodium bisulphite; 100 μL was injected into a high‐performance liquid chromatography system with electrochemical detection (HPLC/ED) with a sodium phosphate mobile phase with l‐octane sulphonic acid as described previously (Hortnagl et al. 2020; Blesa et al. 2012). For determination of dopamine, serotonin, 3,4‐dihydroxyphenylacetic acid and homovanillic acid, 100 μL of the 16,100×*g* supernatant was injected directly into an HPLC/ED system with a sodium phosphate mobile phase with l‐heptane sulphonic acid as described previously (Hortnagl et al. 2020; Blesa et al. 2012).

### 2.5 Quantification of TH Positive Cells in Substantia Nigra Compacta by Immunohistochemical Staining

Paraffin embedded midbrain was cut in 20‐μm slices. Every fifth slice was incubated with monoclonal anti‐TH antibody (1:2000, Boehringer‐Mannheim, Mannheim, Germany) o/n at room temperature. After washing with TRIS buffered saline (TBS), slices were incubated with secondary antibody (antimouse‐alkaline phosphatase antibody, 1:200, Jackson Immuno Research, United States). Slices were washed with TBS and developed with Fast Red reagent, development was stopped with tap water and nuclei were counterstained using haemalaun. Stained sections were photographed (30X magnification), pictures of one slice were combined to one picture automatically using software Autopano Giga 2.5 and substantia nigra compacta was delineated in the pictures according to Nelson et al. (1996). TH+ cells with visible nuclei were counted in a blinded manner.

### 2.6 Immunofluorescent Double Labelling

For the fluorescent double labelling of mouse anti‐Tyrosine hydroxylase (TH, Boehringer Mannheim 1:5000) and rat antihuman α‐synuclein (α‐Syn, Enzo ALX‐804‐258, clone 15G7 1:100), the Akoya Fluorescent Opal dyes were used according to manufacturer's protocol. Sections were deparaffinized in xylene twice for 15 min and rehydrated through graded ethanol to deionized water.

Paraffin imbedded midbrain sections were steamed in citrate buffer (pH 6.0) for 60 min using a household food steamer (Multigourmet FS 20, Braun). Then, a 10‐min blocking step with Opal Antibody Diluent/Blocking Solution (Akoya Biosciences Inc. #ARD1001EA, ready to use) was performed followed by incubation of the primary antibody TH diluted in Opal Antibody diluent overnight at 4°C. Sections were rinsed in Tris‐buffered saline with Tween 20 (TBST, pH 7.5). Secondary antibody antimouse conjugated to horseradish peroxidase (Jackson ImmunoResearch #715‐035‐151 1:200) was incubated for 30 min at room temperature. After washing with TBST, slides were incubated with Opal fluorophore 520 (Akoya Biosciences Inc. #SKU FP1487001KT 1:300). Before introducing the next primary antibody, α‐Syn sections were steamed again using AR6 (Akoya Biosciences Inc. #AR600250ML) for 30 min. After blocking step, α‐Syn was incubated over night at 4°C. Detection was done with secondary antibody antirat conjugated to horseradish peroxidase (ImmunoResearch #712‐035‐153 1:200) and Opal 570 (Akoya #FP1488001KT 1:300). Finally, sections were counterstained with 4′,6‐diamidino‐2‐phenylindone (DAPI). Slides were scanned on a laser scanning microscope (Leica SP5, Mannheim, Germany).

### 2.7 Statistics

Locomotor behaviour was analysed by two‐way analysis of variants for the factors α‐synuclein and nicotinic α7 acetylcholine receptor for the dependent variable ambulatory, nonambulatory or vertical movements accumulated from time 15–30 min after the mice were placed in the activity cage. Monamine neurotransmitters and metabolites were analysed by two‐way analysis of variants (ANOVA) for the factors mutated α‐synuclein and nicotinic α7 acetylcholine receptor for the dependent variable noradrenaline (NA), dopamine (DA), serotonin (5‐HT), 5‐hydroxyindoleacetic acid (5‐HIAA), 3,4‐dihydroxyphenylacetic acid (DOPAC), homovanillic acid (HVA) or DA turnover. DA turnover was calculated by the molar ratio of DOPAC/DA and HVA/DA for each individual mouse. The normality test was performed according to Shapiro–Wilk and the equal variance test according to Brown–Forsythe, the pairwise multiple comparison procedures according to the Holm–Sidak method. Data in graphs and tables show the mean values ± standard error of the mean (SEM). *p* values lower than 0.05 were considered as indicating statistical significance. ANOVA results were reported in the Supporting Information.

## 3 Results

Four experimental groups of mice were investigated in this study: mice wild type for both, α‐synuclein and α7‐acetylcholine receptors; mice wild type for α‐synuclein and an α7‐acetylcholine receptor knockout; mice overexpressing a double‐mutated form of human α‐synuclein under the tyrosine hydroxylase promotor and with α7‐acetylcholine receptors; and mice with the double‐mutated form of human α‐synuclein without α7‐acetylcholine receptors. Three distinct motor features were determined at 7 and 16 months of age. The respective counts accumulated from time 15–30 min after placing the mouse into the activity cage were analysed by ANOVA for the factors mutated α‐synuclein and α7 acetylcholine receptors.

**FIGURE 1:**
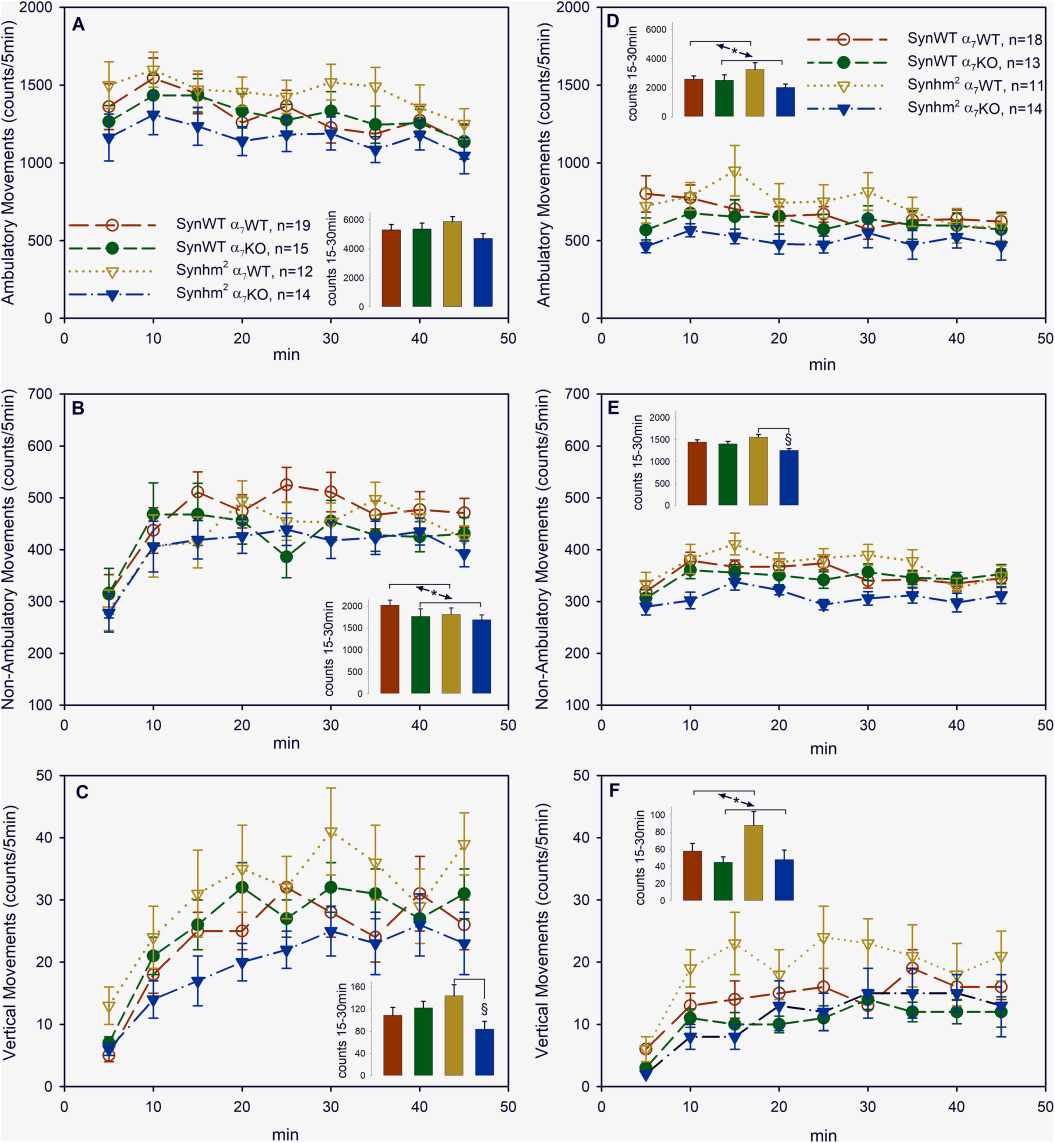
Motor behaviour at 7 (A–C) and 16 (D–F) months of age. Mice with wild‐type α‐synuclein (SynWT, circles) or transgenic for a double mutated human α‐synuclein (Synhm^2^, triangles) and wild‐type nicotinic acetylcholine receptors (α_7_WT, open symbols) or with a knockout of the α_7_ nicotinic acetylcholine receptor (α_7_KO, closed symbols) were analysed for ambulatory movements (A,D), nonambulatory movements (B,E) and vertical movements (C,F) at the times indicated after placing them in an activity metre. The counts accumulated from time 15–30 min are shown as insets (SynWT α_7_WT, red; SynWT α_7_KO, green; Syn hm^2^ α_7_WT, ocher; and Syn hm^2^ α_7_KO, blue) and were analysed by two‐way ANOVA for the factors mutated α‐synuclein and α_7_ acetylcholine receptors; *significantly less counts in α_7_‐knockout mice than in α_7_‐wild‐type mice (*p* < 0.05); ^§^significantly less counts in α_7_‐knockout mice than in α_7_‐wild‐type mice if they contain the mutated α‐synuclein (*p* < 0.05) but not the wild‐type α‐synuclein (*p* > 0.53). Shown are mean values ± SEM.

At 7 months of age, there was no statistically significant difference in ambulatory movements (Figure 1A). For nonambulatory movements, however, two‐way ANOVA showed no statistically significant interaction between α‐synuclein and α7 (*p* = 0.883), but the pairwise multiple comparison procedures of the Holm–Sidak method revealed significantly less nonambulatory movements in α7‐knockout mice than in α7‐wild‐type mice (*p* = 0.048; Figure 1B). In vertical movements, the two‐way ANOVA calculated a statistically significant interaction between synuclein and α7‐receptors (*p* = 0.021) and Holm–Sidak revealed significantly fewer vertical movements in α7‐knockout mice than α‐7‐wild‐type mice if they contain the mutated α‐synuclein (*p* = 0.012) but not in mice with just the mouse wild‐type α‐synuclein (*p* = 0.530; Figure 1C).

At 16 months of age, two‐way ANOVA analysis resulted for ambulatory movements in a statistically significant difference among the levels of α7‐acetylcholine receptors, however no interaction between α‐synuclein and α7, so the pairwise multiple comparison procedures calculated significantly less counts in α7‐KO mice than α7‐wild‐type mice (*p* = 0.042; Figure 1D). On the other hand, also at 16 months of age, there was a statistically significant interaction between the factors synuclein and α7‐receptors, namely, for nonambulatory movements (*p* = 0.020): α7‐KO mice showed significantly less of these stereotypic movements than α‐7‐wild‐type mice if they contain the mutated α‐synuclein (*p* < 0.001) but not just wild‐type mouse synuclein (*p* = 0.552; Figure 1E). Finally, for vertical movements, the statistical analysis yielded similar results as for ambulatory movements: a statistically significant difference among the levels of α7‐acetylcholine receptors (*p* = 0.017) but no interaction between α‐synuclein and α7‐receptor (*p* = 0.227), which means α7‐KO mice showed generally fewer vertical movements than α7‐wild‐type mice (Figure 1F).

At 21 months of age, mice were analysed for striatal tissue levels of monoamine neurotransmitters and dopamine metabolites, as well as for numbers of cells positive for tyrosine hydroxylase in the substantia nigra.

**FIGURE 2:**
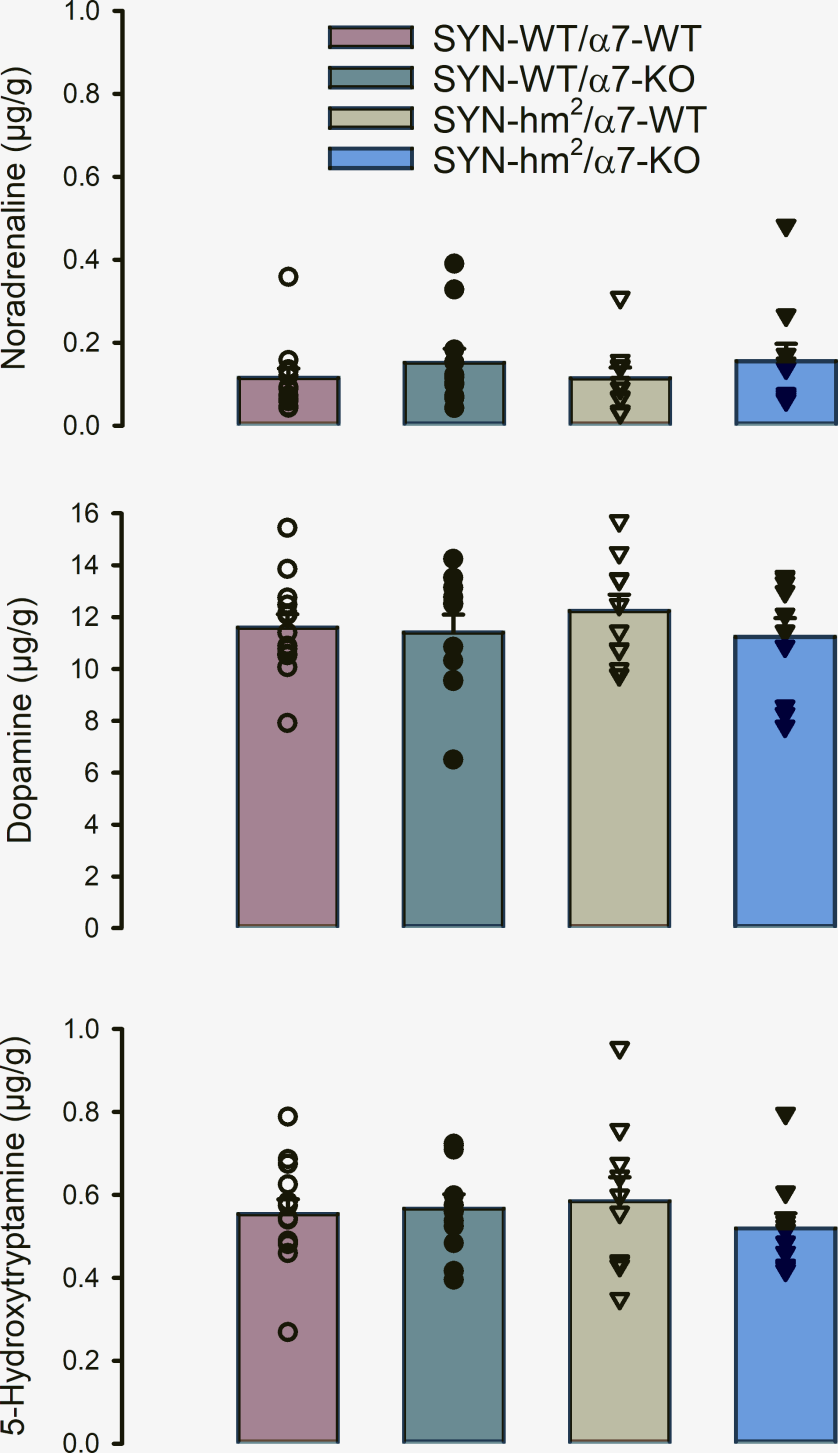
Striatal monoamine neurotransmitter tissue levels at 21 months of age. Mice with wild‐type α‐synuclein (circles) or transgenic for a double mutated human α‐synuclein (triangles) and wild‐type nicotinic acetylcholine receptors (open symbols) or with a knockout of the α7 nicotinic acetylcholine receptor (closed symbols). Shown are single values (*n* = 10–13) and mean values ± SE.

**FIGURE 3:**
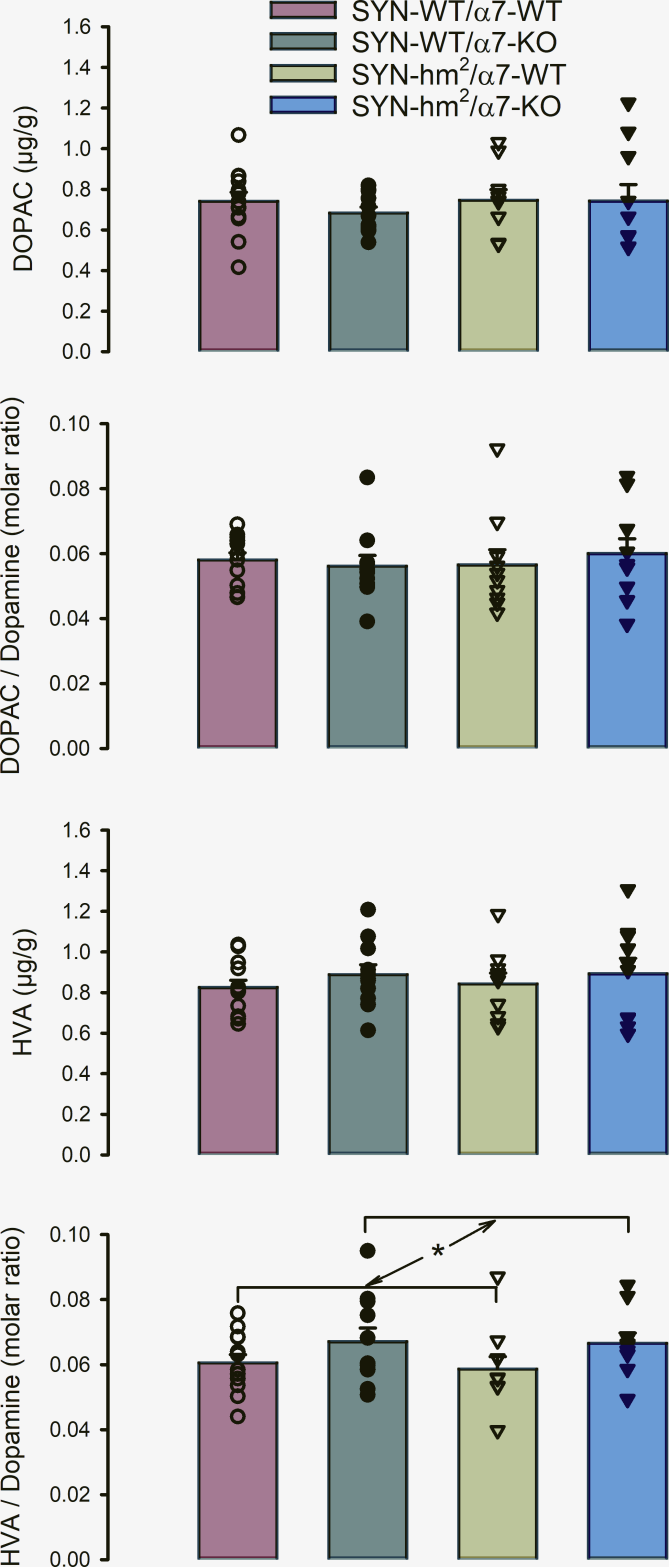
Striatal tissue levels of dopamine metabolites and dopamine turnover at 21 months of age. Mice with wild‐type α‐synuclein (circles) or transgenic for a double mutated human α‐synuclein (triangles) and wild‐type nicotinic acetylcholine receptors (open symbols) or with a knockout of the α7 nicotinic acetylcholine receptor (closed symbols). *Two‐way analysis of variants for the factors SYN and α7 for the dependent variables DOPAC, DOPAC/DA molar ratio, HVA or HVA/DA molar ratio and according Pairwise Multiple Comparison Procedures (Holm–Sidak method) HVA/dopamine is generally higher in α7‐KO than in α7‐WT mice (*p* = 0.041). Shown are single values (*n* = 10–13) and mean values ± SE.

Monoamine neurotransmitter analysis displayed the typical concentration range in the striatum with mean values of dopamine 80 times and serotonin four times higher than that of noradrenaline but without any statistically significant differences due to mutated α‐synuclein or α7‐receptor (Figure 2). There were no statistically significant differences for the levels of the serotonin metabolite 5‐HIAA and serotonin turnover 5‐HIAA/5‐HT either (data not shown). Analysis of the dopamine metabolites yielded somewhat more differentiated results (Figure 3): Two‐way ANOVA for the factors mutated α‐synuclein and α7‐receptor calculated no significant differences for DOPAC, DOPAC/DA or HVA. However, for molar ratios of HVA/dopamine, calculated for each individual mouse, a parameter for dopamine turnover, two‐way ANOVA displayed no interaction between mutated α‐synuclein and α7 (*p* = 0.835), but Holm–Sidak yielded that HVA/dopamine is generally higher in α7‐KO than in α7‐wild‐type mice (*p* = 0.041).

**FIGURE 4:**
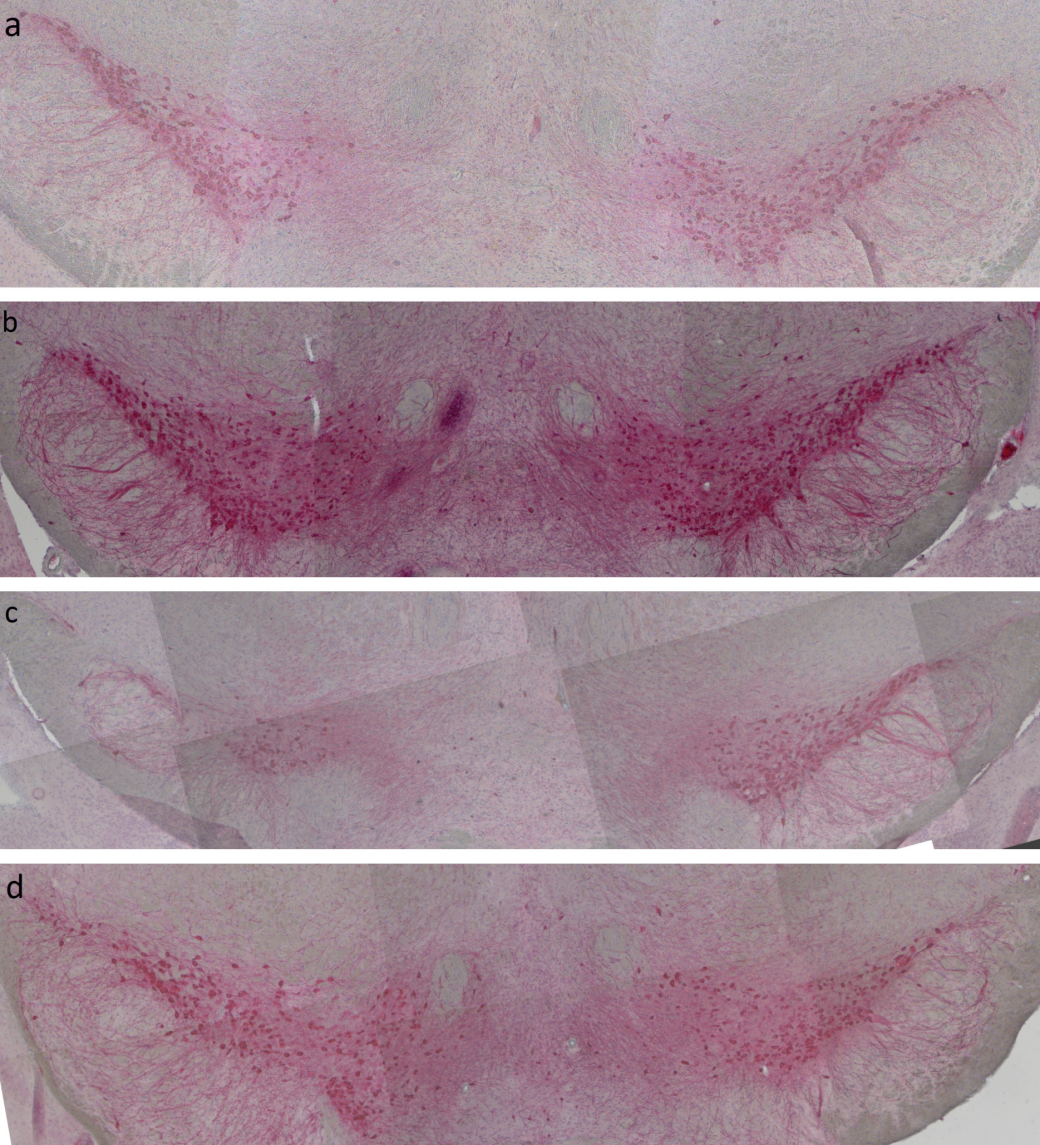
Representative midbrain sections from mice with wild‐type α‐synuclein (a,c) or transgenic for a double mutated human α‐synuclein (b,d) and wild‐type nicotinic acetylcholine receptors (a,b) or with a knockout of the α7 nicotinic acetylcholine receptor (c,d) stained for tyrosine hydroxylase at 21 months of age.

**FIGURE 5:**
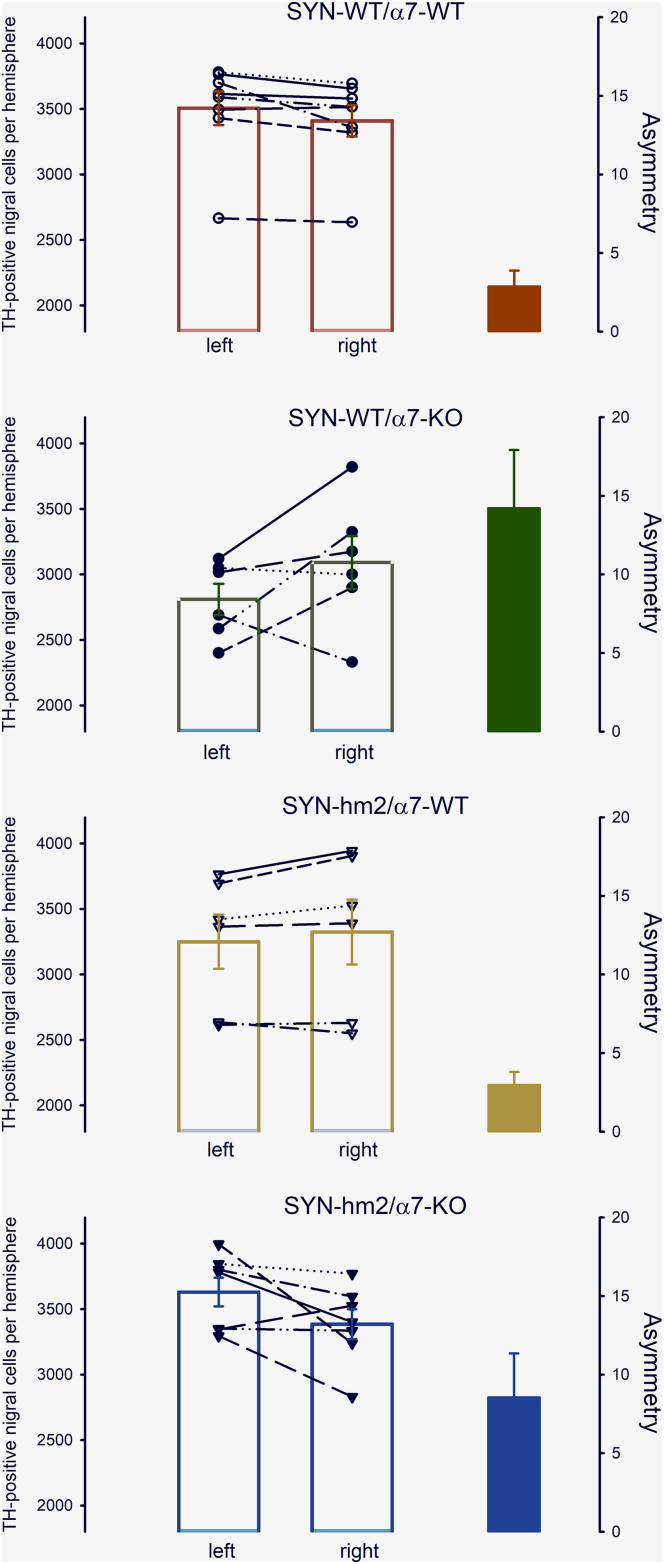
Stereological counts of TH‐positive nigral cells in the left and right hemisphere (open columns) and asymmetry of the distribution (filled columns) as calculated by the absolute value of (cells left minus cells right)/(mean value of cells left and right)%. Two‐way analysis of variants for the factors SYN and α7 for the dependent variable asymmetry; according Pairwise Multiple Comparison Procedures (Holm–Sidak method), the asymmetry in a7‐KO mice is significantly higher than in a7‐WT mice (*p* = 0.001). Shown are single values (*n* = 6–8) and mean values ± SE.

Paraffin‐embedded midbrains were cut in 20‐μm slices and stained for tyrosine hydroxylase. Representative coronal slices (Figure 4) revealed a peculiar asymmetry in the number of TH‐positive neurons in the substantia nigra of mice without α7‐nicotinic acetylcholine receptors (Figure 4c,d), irrespective of the absence (Figure 4a,c) or presence of the mutated α‐synuclein (Figure 4b,d). There was no side preference; TH‐positive neurons could be reduced in the left or right hemisphere. Unbiased stereological counts of all TH‐positive cells in the left and the right substantia nigra compacta were determined in a blinded manner. Asymmetry of the distribution was calculated for each individual mouse by the absolute value of (total cells left minus total cells right) divided by (mean value of total cells left and right). Two‐way analysis of variants for the factors mutated c and α7 for the dependent variable asymmetry revealed that the difference in the mean values of asymmetry among the different levels of α7 is greater than would be expected by chance after allowing for effects of differences in SYN (Figure 5). There is not a statistically significant interaction between mutated α‐synuclein and α7 (*p* = 0.226). Pairwise multiple comparison procedures of the Holm–Sidak method give that asymmetry is generally higher in α7‐KO than in α7‐wild type (*p* = 0.001).

**FIGURE 6:**
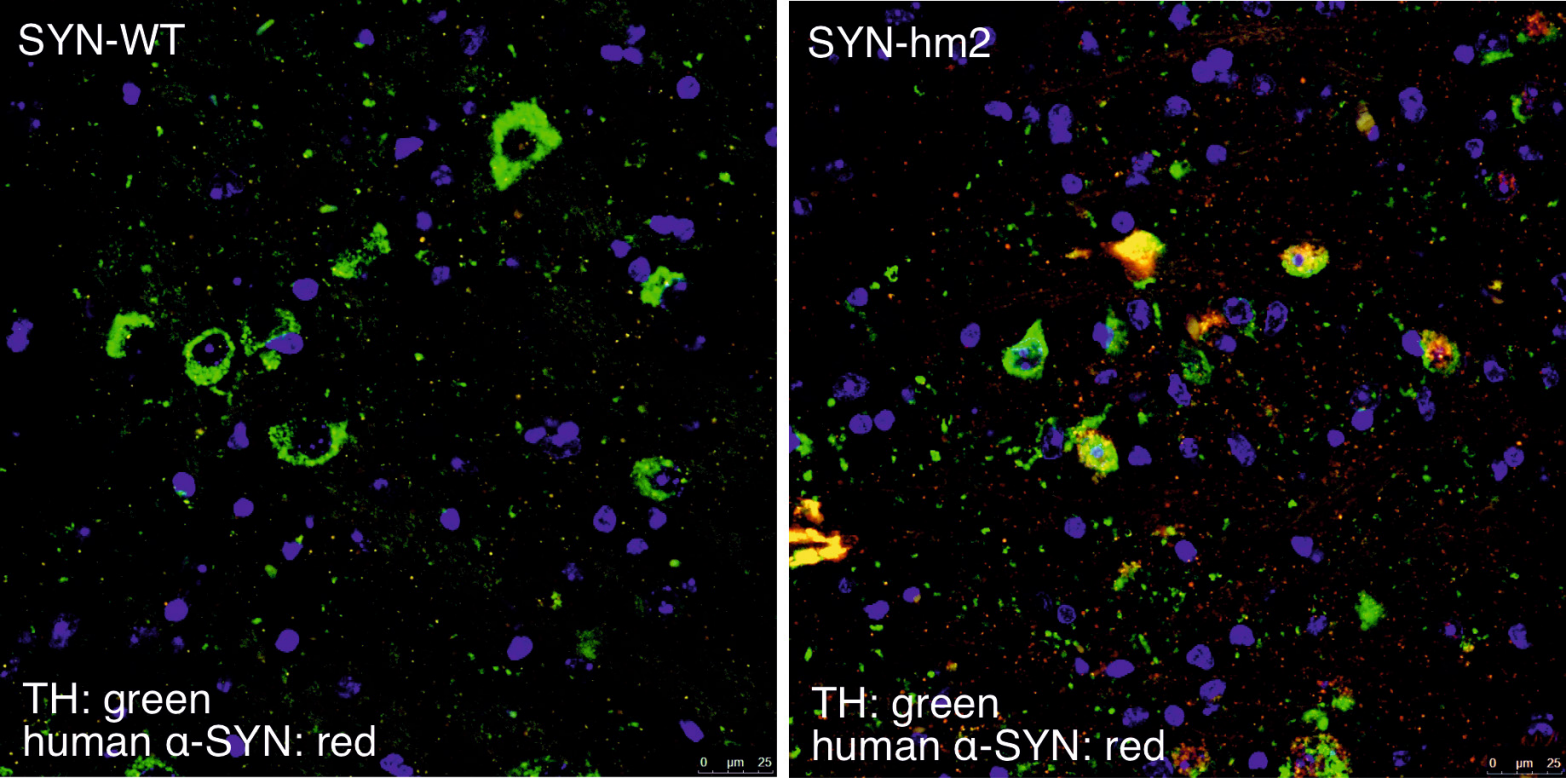
Representative midbrain sections from mice with wild‐type α‐synuclein or transgenic for a double mutated human α‐synuclein were stained at 21 months of age for tyrosine hydroxylase (green) and an antibody specific for human α‐synuclein (red staining), the overlay resulting in a yellow signal.

In additional experiments, some of the paraffin sections were also stained in immunofluorescent double labelling experiments to verify, if the human transgenic α‐synuclein is indeed expressed in midbrain dopaminergic TH‐positive neurons. As can be seen in Figure 6 in both SYN‐WT and SYN‐hm2 slices, several TH‐positive cells can be seen (green staining). Only in slices from SYN‐hm2 transgenic mice some, but not all of the TH‐positive cells can also be stained with an antibody specific for the transgenic human α‐synuclein (red staining), the overlay resulting in a yellow signal.

## 4 Discussion

The main finding of our study is that absence of the α7‐nicotinic acetylcholine receptor impairs motor features in mice overexpressing a double mutated human α‐synuclein but not in wild‐type mice, which express just normal mouse α‐synuclein. Each of these mutations in human α‐synuclein, A53T and A30P cause familial Parkinson's disease with the characteristic motor impairment of this neurodegenerative disorder (Polymeropoulos et al. 1997; Kruger et al. 1998). Thus, our data suggest that the neuroprotective effect of smoking might at least partly be mediated by the nicotine in the cigarettes acting via α7‐acetylcholine receptors. This assumption is in line with previous studies, showing that Varenicline, which is not only a partial agonist on α4β2‐ but also a full agonist on α7‐acetylcholine receptors and a clinically highly efficient drug for smoking cessation therapy, protects efficiently against locomotor alteration in the MPTP mouse model of PD (Ribeiro‐Carvalho et al. 2021). These findings could, however, not be reproduced in humans, where varenicline did not have a positive effect on balance or cognition in PD patients (Kapur et al. 2021); in a different study, it did however significantly improved axial symptoms and rapid alternating movements in patients with spinocerebellar ataxia (SCA) 3 (Zesiewicz et al. 2012).

In our study, the presence of the α7‐nicotinic acetylcholine receptor appears to protect the mice from mutated α‐synuclein, which did not induce overt parkinsonism in mice without the α7‐nicotinic acetylcholine receptors but some impairment in their motor abilities. Deficits in rearing behaviour—which was determined by vertical movements in our open field setup—were reported to correlate with striatal dopamine depletion in mice exposed to the neurotoxin MPTP, which also reduced distance of locomotion, a parameter related to ambulatory movements in our study (Schwarting et al. 1999). That reduction of locomotion and rearing is related to an impact on the dopamine system is supported by reduced levels of locomotion and rearing in dopamine receptor deficient mice (Wang et al. 2000) and the reinstating effect of L‐Dopa in MPTP treated mice (Fredriksson and Archer 1995). Within the various motor features, rearing behaviour seems to be particularly sensitive to changes in dopamine function (Willard et al. 2015). In fact, the per cent reduction induced by knockdown of the α7‐nicotinic acetylcholine receptor and by the expression of the mutated α‐synuclein was highest in the parameter of vertical movements, which reflect rearing behaviour in our study. However, we did not observe a dopamine depletion in our mice. What we observed is an increased molar ratio of HVA/dopamine, a measure of increased dopamine turnover, in mice with gene deletion of the nicotinic α7‐acetylcholine receptor. These mice showed impairment in their motor abilities if they additionally expressed the mutated α‐synuclein. If the reduced nonambulatory movements are due to dopamine receptors desensitized by the increased turnover, it can only be speculated, but nonambulatory is closely related to stereotypic behaviour, which depends on dopamine receptor sensitivity (Hinzen et al. 1986; Klawans et al. 1985).

Increased dopamine turnover is quite common in animal models after an impact on the dopamine system but still void of a plain dopamine loss. Mice exposed to a combination of paraquat and maneb displayed a reduced locomotor activity and an increased striatal dopamine turnover with still unchanged dopamine levels, a treatment resulting in significantly reduced tyrosine hydroxylase density after repetitive administration (Thiruchelvam et al. 2000). Striatal dopamine turnover increased in mice with unchanged dopamine concentration following acute administration, whereas mice had reduced striatal dopamine after repeated administration of rotenone (Thiffault et al. 2000; Inden et al. 2007). The epidemiological linkage between insecticide exposure and the incidence of Parkinson's disease prompted the effects of chlorpyrifos on dopamine pathways in mice in which rearing was decreased, striatal dopamine levels remained unchanged but dopamine turnover was increased (Karen et al. 2001). Mutated genes, which can lead to familial Parkinson's, are also associated with increased dopamine turnover without or before manifest nigrostriatal dopamine loss. Whereas LRRK2 mutation carriers with Parkinson's disease reveal impaired presynaptic dopamine function (Nandhagopal et al. 2008), clinically asymptomatic, otherwise, healthy, mutations carriers reveal early increases in dopamine turnover by PET imaging (Sossi et al. 2010). In mice knockout of DJ‐1, which induces autosomal recessive early‐onset parkinsonism by homozygous mutations in humans, increased dopamine turnover (Raman et al. 2013) and knock‐in of the vascular protein sorting 35 with a mutation linked to dominantly‐inherited, late‐onset parkinsonism in humans resulted in normal dopamine levels in striatal tissue but increased dopamine turnover as determined by microdialysis in the dorsolateral striatum (Cataldi et al. 2018). Just as in these reports, neurotoxins and genetic factors increased dopamine turnover in a first step before overt nigrostriatal degeneration; the increased dopamine turnover in α7‐knockout mice could be evidence of an impaired nigrostriatal system that responds to mutant α‐synuclein with motor dysfunction.

Another indication of an impaired nigrostriatal system in α7‐knockout mice could be that their substantia nigra contains fewer TH‐positive neurons in one of the hemispheres than in the other, a finding reflected by a statistically significant increase in asymmetry in the number of TH‐positive neurons. There was no side preference so that TH‐positive neurons were observed slightly reduced in the left or right hemisphere. Evidence of asymmetry differentiates Parkinson's disease from other neurodegenerative parkinsonian syndromes (Gelb et al. 1999). There is greater neuronal loss in the substantia nigra contralateral to the initially more affected side (Kempster et al. 1989). Even in hereditary cases with mutations in α‐synuclein, *DJ‐1*, *PINK* and *LRKK2* genes' unilateral presentation is common, and there is no side preference in the members of the affected families (Djaldetti et al. 2006). Also, in the established example of an exogenous cause of parkinsonism due to MPTP exposure symptom, onset was frequently unilateral at disease on set in these patients (Ballard et al. 1985). There are only few studies in mouse models of Parkinson's disease on the asymmetry of neuropathological changes. However, repeated oral administration of rotenone resulted in an increased asymmetric grade calculated by large numbers/small number of TH‐immunopositive neurons in any right and left substantia nigra (Inden et al. 2007) and intraperitoneal paraquat injections every second day over 2 weeks induced hemispheric differences in the substantia nigra as determined by high‐resolution magnetic resonance (Dwyer et al. 2021).

On these lines, our results suggest that the nigrostriatal dopamine system relies on the presence of α7 nicotinic acetylcholine receptors to ensure normal dopamine turnover and viable dopamine neurons in the substantia nigra, where increased dopamine turnover via oxidized dopamine could trigger a feedforward mechanism leading to the death of additional neighbouring dopamine neurons (Burbulla et al. 2017) and ultimately an asymmetric dopamine cell loss initially triggered by these deleterious cellular pathways stochastically in one of the hemispheres. Against this background of a partially impaired nigrostriatal dopamine system, mutant α‐synuclein appears to induce motor deficits without overt additional neurochemical or neuropathological changes. Similar to how physiological stimulation of nicotinic acetylcholine receptors in wild‐type mice protects against this scenario, the neuroprotective effect of smoking could be explained by an additional nicotine effect on these receptors.

## Author Contributions

**Christian Pifl:** conceptualization, supervision, writing – original draft. **Alexandra Wolf:** data curation. **Mark Elevado:** data curation. **Petra Scholze:** conceptualization, funding acquisition, writing – original draft.

## Ethics Statement

All animal experiments were performed in accordance with EU (Directive 210/63/EU) and Austrian federal law (Tierversuchsgesetz 2012) after obtaining approval from the animal subjects review board of the Medical University of Vienna and the Austrian Federal Ministry of Education, Science and Research (GZ: 2022‐0.638.952).

## Conflicts of Interest

The authors declare no conflicts of interest.

### Peer Review

The peer review history for this article is available at https://www.webofscience.com/api/gateway/wos/peer‐review/10.1111/ejn.70063.

## Supporting information


**Table S1** Breeding scheme of transgenic animals used in the study

## Data Availability

The datasets used and/or analysed during the current study are available from the corresponding author on reasonable request.
