# Supplementary material for: α7‐Nicotinic Acetylcholine Receptor and Mutated α‐Synuclein Interact in Motor Behaviour and Nigrostriatal Dopamine—Findings With Potential Relevance for a Protective Effect of Cigarette Smoking and Parkinson's Disease
Source: Eur J Neurosci. 2025 Mar 24;61(6):e70063. doi: 10.1111/ejn.70063 (PMC11931489; doi:10.1111/ejn.70063)
Supplement: Supplementary file 1 — Table S1 Breeding scheme of transgenic animals used in the study [file EJN-61-0-s001.pdf]

Supplementary information

α7-nicotinic acetylcholine receptor and mutated α-synuclein interact in motor behavior and nigrostriatal dopamine, findings with potential relevance for a protective effect of cigarette smoking and Parkinson's disease

Christian Pifl, Alexandra Wolf, Mark Elevado and Petra Scholze

Supplemental table 1: Breeding scheme of transgenic animals used in the study

Generation	parent 1	parent 2
F0	α7-/α7-<br>Syn-WT/ Syn-WT	α7+/α7+<br>Syn-WT /Syn-hm2
	offspring	
F1	α7+/α7-<br>Syn-WT/ Syn-WT	α7+/α7-<br>Syn-WT /Syn-hm2

Generation	parent 1		parent 2	
F1	α7+/α7-<br>Syn-WT/ Syn-WT		α7+/α7-<br>Syn-WT /Syn-hm2	
	offspring			
F2	α7+/α7+<br>Syn-WT/ Syn-WT	α7-/α7-<br>Syn-WT/ Syn-WT	α7+/α7+<br>Syn-WT /Syn-hm2	α7-/α7-<br>Syn-WT /Syn-hm2

ANOVA and Pairwise Multiple Comparison Procedures results

Non-ambulatory movements at 7 months of age

Source of Variation	DF	SS	MS	F	P
SYN	1	213854.985	213854.985	0.778	0.381
a7	1	1123537.591	1123537.591	4.088	0.048
SYN x a7	1	6045.925	6045.925	0.0220	0.883
Residual	57	15667206.721	274863.276		
Total	60	17195309.016	286588.484		

Vertical movements at 7 months of age

Source of Variation	DF	SS	MS	F	P
SYN	1	21.797	21.797	0.00627	0.937
a7	1	8117.902	8117.902	2.335	0.132
SYN x a7	1	19523.991	19523.991	5.615	0.021
Residual	57	198206.558	3477.308		
Total	60	223517.639	3725.294		

Pairwise Multiple Comparison Procedures (Holm-Sidak method):
Comparisons for factor: **a7 within SYN-hm2**

Comparison	Diff of Means	t	P	P<0.050
a7-WT vs. a7-KO	59.633	2.611	0.012	Yes

Ambulatory movements at 16 months of age

Source of Variation	DF	SS	MS	F	P
SYN	1	99499.653	99499.653	0.0734	0.787
a7	1	5867434.616	5867434.616	4.330	0.042
SYN x a7	1	4476398.163	4476398.163	3.303	0.075
Residual	52	70464107.337	1355078.987		
Total	55	79866622.638	1452120.412		

Pairwise Multiple Comparison Procedures (Holm-Sidak method):
Comparisons for factor: **a7**

Comparison	Diff of Means	t	P	P<0.050
a7-WT vs. a7-KO	657.612	2.081	0.042	Yes

Non-ambulatory movements at 16 months of age

Source of Variation	DF	SS	MS	F	P
SYN	1	3384.087	3384.087	0.0861	0.770
a7	1	401206.470	401206.470	10.210	0.002
SYN x a7	1	224986.900	224986.900	5.726	0.020
Residual	52	2043342.824	39295.054		
Total	55	2633812.554	47887.501		

Pairwise Multiple Comparison Procedures (Holm-Sidak method):
Comparisons for factor: **a7**

Comparison	Diff of Means	t	P	P<0.050
a7-WT vs. a7-KO	171.961	3.195	0.002	Yes

Comparisons for factor: **a7 within SYN-hm2**

Comparison	Diff of Means	t	P	P<0.050

a7-WT vs. a7-KO	300.734	3.765	<0.001	Yes

Vertical movements at 16 months of age

**Source of Variation**	**DF**	**SS**	**MS**	**F**	**P**
SYN	1	3743.929	3743.929	2.350	0.131
a7	1	9499.752	9499.752	5.963	0.018
SYN x a7	1	2380.934	2380.934	1.494	0.227
Residual	52	82847.559	1593.222		
Total	55	96276.062	1750.474		

Pairwise Multiple Comparison Procedures (Holm-Sidak method):
Comparisons for factor: **a7**

**Comparison**	**Diff of Means**	**t**	**P**	**P<0.050**
a7-WT vs. a7-KO	26.461	2.442	0.018	Yes

Molar ratios of HVA/Dopamine

**Source of Variation**	**DF**	**SS**	**MS**	**F**	**P**
SYN	1	0.0000165	0.0000165	0.128	0.722
alpha7	1	0.000571	0.000571	4.452	0.041
SYN x alpha7	1	0.00000564	0.00000564	0.0440	0.835
Residual	40	0.00513	0.000128		
Total	43	0.00572	0.000133		

Pairwise Multiple Comparison Procedures (Holm-Sidak method):
Comparisons for factor: **alpha7**

**Comparison**	**Diff of Means**	**t**	**P**	**P<0.050**
a7-KO vs. a7-WT	0.00725	2.110	0.041	Yes

Asymmetry of TH-positive nigral cells in the left and right hemisphere

**Source of Variation**	**DF**	**SS**	**MS**	**F**	**P**
SYN	1	51.304	51.304	1.436	0.243
a7	1	476.367	476.367	13.332	0.001
SYN x a7	1	55.289	55.289	1.547	0.226
Residual	23	821.799	35.730		
Total	26	1384.264	53.241		

Pairwise Multiple Comparison Procedures (Holm-Sidak method):
Comparisons for factor: **a7**

**Comparison**	**Diff of Means**	**t**	**P**	**P<0.050**
a7-KO vs. a7-WT	8.461	3.651	0.001	Yes
